# Service Use, Charge, and Access to Mental Healthcare in a Private Kenyan Inpatient Setting: The Effects of Insurance

**DOI:** 10.1371/journal.pone.0090297

**Published:** 2014-03-20

**Authors:** Victoria Pattison de Menil, Martin Knapp, David McDaid, Frank Gitau Njenga

**Affiliations:** 1 Department of Social Policy, London School of Economics and Political Science, London, United Kingdom; 2 Personal Social Services Research Unit, London School of Economics and Political Science, London, United Kingdom; 3 Chiromo Lane Medical Centre, Nairobi, Kenya; Supportive care, Early DIagnosis and Advanced disease (SEDA) research group, United Kingdom

## Abstract

The gap in Kenya between need and treatment for mental disorders is wide, and private providers are increasingly offering services, funded in part by private health insurance (PHI). Chiromo, a 30-bed psychiatric hospital in Nairobi, forms part of one of the largest private psychiatric providers in East Africa.

The study evaluated the effects of insurance on service use and charge, questioning implications on access to care. Data derive from invoices for 455 sequential patients, including 12-month follow-up. Multi-linear and binary logistic regressions explored the effect of PHI on readmission, cumulative length of stay, and treatment charge.

Patients were 66.4% male with a mean age of 36.8 years. Half were employed in the formal sector. 70% were admitted involuntarily. Diagnoses were: substance use disorder 31.6%; serious mental disorder 49.5%; common mental disorder 7%; comorbid 7%; other 4.9%. In addition to daily psychiatric consultations, two-thirds received individual counselling or group therapy; half received lab tests or scans; and 16.2% received ECT. Most took a psychiatric medicine. Half of those on antipsychotics were given only brands.

Insurance paid in full for 28.8% of patients. Mean length of stay was 11.8 days and, in 12 months, 16.7 days (median 10.6). 22.2% were readmitted within 12 months. Patients with PHI stayed 36% longer than those paying out-of-pocket and had 2.5 times higher odds of readmission. Mean annual charge per patient was Int$ 4,262 (median Int$ 2,821). Insurers were charged 71% more than those paying out-of-pocket - driven by higher fees and longer stays.

Chiromo delivers acute psychiatric care each year to approximately 450 people, to quality and human rights standards higher than its public counterpart, but at considerably higher cost. With more efficient delivery and wider insurance coverage, Chiromo might expand from its occupancy of 56.6% to reach a larger population in need.

## Introduction

Government allocations account for only one third (30.0%) of Kenyan health spending. Two-thirds of the Int$78 per capita health expenditure [1] are split between international donors (29.4%) and out-of-pocket payments (36.7%), with the remainder from private companies [2]. Out-of-pocket payments (OPP) go predominantly (76.3%) to hospitals, including private for-profit hospitals, which account for 14.9% of this expenditure (ibid). OPP are associated with catastrophic loss in low-income countries [3], so policy makers have been vying to create social health insurance [4], [5].

In 2004, Kenya's parliament passed a promising bill to create a National Social Health Insurance Fund, which would fund both outpatient and inpatient care for all Kenyans using a sliding scale of contributions [4]. Disappointingly, the bill was not signed into law, because of concern over the feasibility of its financing. Now, the only operational social insurance is the National Hospital Insurance Fund (NHIF), which is under investigation by Kenya's Ethics and Anti-Corruption Commission, and which allocates only 22% of funds towards benefits [4]. NHIF pays a flat-fee of Ksh 800 (Int$ 20.8) for inpatient stays and enrolment is mandatory for all formal sector employees; but currently it covers only 5.5% of the population [2].

In this context of barriers to national insurance, private health insurance (PHI) remains one alternative to user fees for financing healthcare, among those who can afford it. In Kenya, PHI is used by 2% of the population and accounts for 4% of total health expenditure [2], [6]. Critics of PHI argue that it benefits only the rich and leads to spiralling use and costs of services, while proponents suggest that it provides financial protection, increases early access to services, and mitigates problems of wait-time and quality [7]. The debate remains largely theoretical in low-income settings in the absence of evidence. A systematic review of randomised controlled trials and observational studies about the impact of health insurance in Africa and Asia found only one study of PHI, from Asia [8].

Despite broad interest in private healthcare in Africa [9], there is also a noted gap in the literature on private mental healthcare [10], [11] with some exception for private-public partnerships [12]. In Kenya, mental health is among the lowest priorities of the public health system, accounting for less than 1% of the health budget – on par with the mean of 0.5% across low-income countries [13]. Public psychiatric inpatient care for a population of 38 million is relegated to one 600-bed psychiatric hospital, Mathari, seven provincial and six district hospitals with psychiatric wards of approximately 20 beds each.

Private providers are increasingly offering mental health services in Kenya, particularly for substance disorders. Kenya has 80 practicing psychiatrists and 44% of them work in private practice [14]. Kenya's national authority on drug and alcohol abuse (NACADA) lists 35 registered rehabilitation centres: only three are public, and 80% were founded since 2000 [15]. While no local studies have offered an explanation for this growth, evidence from rapidly developing countries in Asia suggests that, on the demand-side, service users prefer non-state provision because they perceive the non-state sector as having more flexible access, shorter waiting times, greater confidentiality, and greater sensitivity to their needs [16]. Anecdotally, psychiatric patients in Kenya cite greater availability and wider choice of medication as an incentive for private care. On the supply-side, Kenya's Mental Health Act of 1989 prohibits discrimination against mental illness by insurance companies. In recent years, most insurance companies have changed their policies to obey this law. AAR was a leader in that change, under the chairmanship of Frank Njenga, co-author on this paper. In addition, substantial growth of the Kenyan economy in recent years is likely to expand the number of citizens who are in a position to obtain private health insurance [17], [18].

This paper looks in detail at one private facility, the Chiromo Lane Medical Centre. Founded in 1996, Chiromo offers acute private psychiatric care in a small (30-bed) hospital with comfortable accommodation, aspiring to “recovery in dignity.” Located in a former private home in the wealthy Westlands neighbourhood of Nairobi, it maintains the structure and feel of a home – open doors, small rooms and a garden. Chiromo is part of a five-facility hospital group, consisting of two other acute psychiatric hospitals, a rehabilitation centre and a half-way house for substance abusers. The founding staff are a social worker and three psychiatrists trained at the Maudsley hospital in London, UK, prior to establishment of psychiatric training in Kenya (1971). With a joint capacity of 102 beds, the Chiromo Hospital Group is one of the largest providers of private psychiatric services in Kenya and East Africa.

The purpose of this paper is to evaluate the effects of PHI on 1) the quantity of psychiatric services provided; and 2) total charge, and to explore the implications on access to care.

## Methods

Service utilisation and charge were analysed from all hospital invoices between 28 March 2011 and 27 March 2012. Twelve-month follow-up data was obtained on readmissions. Medication use was recorded for a sub-set of the first 100 unique discharges in the same period. These data were complemented by direct observation of clinical practice by the first author over two weeks in June 2012.

In order to explore variations in amount and components of care, linear and binary logistic regressions were run in SPSS 19 on four dependent variables: readmission, annual length of stay, charge per day, and annual charge. Covariates were age, sex, diagnosis, insurance, and whether the patient was employed.

In costing, the unit bed fee was held constant using March 2012 rates. Int$ were converted using the International Monetary Fund rate of 38.4 Ksh to 1 Int$. Doctors' fees were imputed for one third of discharges using mean values according to doctor and payment type.

The research methodology was approved by the Ethics and Research Committee of Kenyatta National Hospital and University of Nairobi (P450/10/2011). Patient consent for review of medical records was waived by the review committee.

## Results

### 1. Patients

455 unique patients were seen in 12 months.

#### Demographics

Two thirds were male with a mean age of 36.3 years (range 14–82) and nationally representative religions (table 1) [19]. Three-quarters provided addresses in Nairobi; and during the site visit, 20% were foreign, from the Democratic Republic of Congo, Somalia and South Sudan. Half were employed in the formal sector, and a quarter were students.

**Table 1:** Population Characteristics & Services Used.

Demographics:	
Male	66.4%
Age	36.3 (sd 13.6)
Nairobi address	77.7%
Religion:	
Christian	90.6%
Muslim	8.0%
Hindu or undeclared	1.4%
Occupation:	
Formal sector employment	50.6%
Students	26.7%
Unemployed or retired	13.5%
House-wives	4.6%
Farmer or informally employed	4.6%
Earning income	55.2%
Diagnosis:	
Alcohol disorder	21.2%
Drug disorder	10.4%
Schizophrenia or non-affective psychosis	38.4%
Bipolar disorder	11.4%
Schizo-affective disorder	2.7%
Depression or anxiety	7.0%
Comorbid psychiatric disorders	7.0%
Financing:	
Insurance (100% of fees)	15.8%
Employer (100% of fees)	12.7%
Out-of-pocket	71.5%
NHIF (Int$20 co-pay)	29.0%
Services:	
Individual counseling	60.2%
Group therapy	36.9%
Lab tests	55.6%
Scan	9.9%
ECT	16.2%
Psychiatric medicine	91.0%
Medicines	
Antipsychotic	81.0%
Sedatives	70.0%
Antidepressants	21.0%
Anticonvulsants	17.0%
Alcohol or drug medicine	9.0%
Lithium	5.0%
Methylphenidate	2.0%
Received only branded antipsychotics	50.6%
Received an injection	76.7%

Length of stay	mean	median	sd
Length of stay per hospitalization	11.8 days	9.0 days	10.0
Cumulative length of stay in 12 months	16.7 days	10.6 days	18.5
Involuntary admissions	70.0%		

#### Diagnosis

One third had a primary diagnosis of substance use disorder, and half had a serious mental disorder (SMD) (e.g. schizophrenia, bipolar, psychosis). Only 7% had a common mental disorder (CMD) (e.g. depression or anxiety); a further 7% had comorbid psychiatric diagnoses.

#### Insurance

A quarter (28.5%, n = 130) had care paid in full by an insurance provider (n = 72) or employer (n = 58). Twenty-one insurers and 29 companies provided coverage, in all cases without co-payment. There was no association between diagnosis and being insured (chi square p = 0.54). The association between being insured and being employed was not significant at the 0.05 level (chi-square, p = 0.08). In addition, NHIF partially reimbursed 29% of patients (n = 133) with Int$ 20/day (Ksh 800). A third (36.8%) of those receiving NHIF paid for the rest of their care out of pocket.

An interview with one insurer indicated an inpatient coverage ceiling of Int$ 6,600 (Ksh 250,000), which had increased 2.5 fold in the past decade from Int$ 2,600 (Ksh 100,000). Suicidality and substance use disorders were excluded from all coverage; and pre-existing conditions were excluded from individual, as opposed to corporate, coverage. The pre-existing condition policy recently changed, however, to introduce coverage sub-limits for people with chronic disease.

### 2. Service Use

Patients were admitted by their psychiatrist. Fourteen psychiatrists admitted patients in 12 months; however 87% of patients were admitted by only four doctors, three of them hospital directors.

#### Psychological therapies

In addition to daily consultations of approximately 15 minutes with a psychiatrist, two-thirds of patients received either individual counselling (60.2%) or group therapy (36.9%). Also, all patients were invited to participate in daily half-hour community meetings, attended by nursing and counselling staff, which were occasions for voicing “compliments and complaints” about care. Counselling was given by one of four full-time university educated counsellors. Patients receiving individual counselling averaged 1.2 sessions per week, each lasting up to an hour. Group therapy existed in various forms, including psycho-education, art therapy and (rarely) family therapy. Patients receiving group therapy averaged 1.4 sessions a week.

#### Lab tests and scans

Over half of all patients received a lab test or a scan. The most common lab tests were blood tests, liver and thyroid functions, urinalysis, and diagnostics for infectious disease (e.g. HIV, malaria). Tests were also conducted to verify blood levels of medications, such as lithium. Scans included x-rays, electroencephalograms (EEG) for epilepsy, computerised tomography (CT) head scans, magnetic resonance imaging (MRIs) and ultrasounds.

#### Electro-convulsive therapy (ECT)

Sixteen percent (n = 74) received modified ECT, averaging 4.9 sessions (max12, s.d. 1.8).

#### Medicines

Nearly all (91%) patients were prescribed a psychiatric medicine (mean 3.3 medicines). The most commonly prescribed medicines were: antipsychotics 81%; sedatives 70%; antidepressants 21%; and anticonvulsants 17%. A wide range of medicines was available, including six antidepressants (3 selective serotonin reuptake inhibitors, 2 seratonin-neuropinephrine reuptake inhibitors and an adrenergic receptor antagonist) and nine antipsychotics (4 typical, 5 atypical).

In general, doctors prescribed branded drugs, and the pharmacist was required to fill the prescription as written. Six of the nine antipsychotics and five of six antidepressants were prescribed in only brand form. Among patients taking antipsychotics, half (50.6%, n = 42) were given only branded medicines. For olanzapine, given in brand and generic forms, the brand cost 15 times the generic. Reasons for preferring brands included: 1) greater quality assurance; 2) preferable means of administration (e.g. soluble drops for olanzapine); 3) patient preference.

At the site visit, 70% (n = 14) of patients were admitted involuntarily, and therefore had limited treatment choice, including medication. (Kenya's mental health law requires authorization from one doctor and one family member for involuntary admission.) Some said they received injectable medicines more than they would like. Indeed, three-quarters (76.7%) received an injection. Doctors and nurses preferred injection because it circumvents problems of adherence. In the words of one staff: “*If you refuse medicines, what help are you getting? … You are wasting a whole day that is payable.*”

### 3. Length of Stay, Readmission and Charge

#### Length of stay and readmission

Mean length of stay (LOS) was 11.8 days (max 93, sd 10.0). One quarter (22.2%) of patients were readmitted within 12 months (mean 1.4 readmissions). Mean cumulative stay in 12 months was 16.7 days (median 10.6, max 153, sd 18.5).

#### Charges and components of care

Chiromo charged fee-for-service with lower fees for patients paying out-of-pocket. Patients paid a flat “bed fee” per night for accommodation and hired staff, which amounted to nearly half (45.8%) the total charge (table 2). A minority (11.8%) had private rooms, while the remainder stayed in shared rooms for 3–4 people. Those paying out-of-pocket paid an upfront deposit of Int$ 911 (Ksh 35,000) to cover approximately one week of “bed fees.”

**Table 2:** Component and Aggregate Charges.

Mean Component Charge	Int$	Ksh	% total charge
“Bed fee” (hotel fee) - out-of-pocket	117	4,500	45.8%
“Bed fee” - insured	130	5,000	
Psychiatrist	78–104	3,000–4,000	30.2%
Medication per day	32	1,220	10.0%
E.C.T	299	11,500	7.9%
Labs & scans	151	5,800	3.6%
MRI	234	9,000	
x-ray	39	1,500	
Talk therapy			1.6%
art therapy	5	200	
psycho-education groups	13	500	
individual counselling	26	1,000	
Other (eg external consults)			0.9%
**Total**			**100.0%**

Aggregate Charge	Int$	Ksh	S.D.
Mean charge per day	266	10,227	2,625 (Ksh)
Mean charge per year	4,276	164,215	167,927 (Ksh)
Median charge per year	2,821	108,326	71,622 (Ksh)

The second leading component of charges was psychiatric consultations, which represented one third (30.2%) of fees. Medications were the third highest expense, amounting to 10.0% of the invoice. The lowest-cost intervention was non-medical psychological therapies at only 1.6% of charges.

Mean charge per patient day in the general ward was Int$ 266 (Ksh 10,218). The distribution of total annual charge was skewed with mean Int$ 4,262 (Ksh 163,648) and median Int$ 2,821 (Ksh 108,333).

Regression results (table 3) indicate that having PHI was a significant predictor of readmission, cumulative length of stay and charge per day. Patients with PHI were 2.5 times more likely to be readmitted than those paying out-of-pocket, controlling for diagnosis, age, sex, and employment. They also stayed 36% longer (95% CI 13%–60%), and paid on average 25% more per day (95% CI 17%–34%) than those paying out-of-pocket. The combination of longer stays and higher pricing led the overall annual charge to be on average 71% higher (95% CI 35%–117%) for those with PHI than those paying out-of-pocket.

**Table 3:** Predictors of readmission, length of stay and charge (regression analyses).

	Readmission (yes/no) (n = 244)			annual LOS (ln) (n = 244)			Charge/day (ln) (n = 242)			Total charge (ln) (n = 242)		
Predictor	Exp(B)	S.E.	Sig.	B	S.E.	Sig.	B	S.E.	Sig.	B	S.E.	Sig.
Age	0.99	0.02	0.11	0.01	0.01	0.11	0.00	0.00	0.73	0.01	0.00	0.07
Sex	1.14	0.46	0.67	−0.05	0.12	0.67	−0.05	0.03	0.13	−0.10	0.12	0.41
Payer	2.47	0.44	**0.01**	0.31	0.12	**0.01**	0.22	0.04	**<.01**	0.54	0.12	**<0.01**
Diagnosis												
- SMD	1.98	0.52	0.70	0.14	0.12	0.70	0.07	0.03	**0.04**	0.21	0.12	0.08
- CMD & other	1.63	0.70	0.24	0.06	0.16	0.24	0.02	0.05	0.62	0.09	0.16	0.56
Employed	2.22	0.49	**0.06**	−0.21	0.11	**0.06**	−0.03	0.03	0.37	−0.24	0.11	**0.03**
Log likelihood or R^2^	LL = 156			R^2^ = 0.04			R^2^ = 0.15			R^2^ = 0.10		

Reference group for diagnosis is substance use disorder.

Age and sex had no effect on service use outcomes (length of stay, readmission, charge); however their effects may have been mediated by diagnosis and employment. Being employed was associated with lower rates of readmission and shorter lengths of stay; while having a severe mental disorder was associated with a slightly higher charge per day. Running the regression for readmission without employment and with a larger sample (n = 301) showed that severe mental disorder was significantly associated with readmission. It seems that the effect of having a severe mental disorder is mediated by a greater likelihood of not being in paid employment, which itself appears to shorten the overall hospital stay.

## Discussion

The salient finding of this study is the positive association between PHI and quantity of care. In the absence of data on health outcomes, it cannot be rigorously determined whether additional days of hospitalization and readmission indicate moral hazard of insurance or greater access to needed treatments; however the literature provides some benchmarking of quantity of care against which to begin to assess the quality of care in this setting.

Literature from other parts of the world on the effects of insurance on service use for chronic disease suggest that insurance may be associated with more care. On the side of quantity, a small study from Argentina found the odds of anti-depressant use were 7.2 times higher among the insured than the uninsured [20]. A study examining insurance effects among 3,824 participants with common mental disorders in Santiago, Chile, found that half were privately insured, and they had 2.7 times higher odds of receiving a mental health consultation than those publicly insured, adjusting for the more severe symptoms found among the publicly insured [21]. Health insurance was therefore associated with more coverage in a context of low overall coverage (20% of those with disorders received any consultation), but also with increased inequality. Health outcomes for those on insurance may also be better, as suggested by findings from a large-scale study of a general population in China where having health insurance (of unspecified type) was associated with lower severity of depressive symptoms [22].

In a higher-income context, the inverse relationship was found in relation to quantity of care and private insurance. A study from Israel observed that when insurance was associated with fee-for-service (ie a fee per inpatient day), it resulted in lower length of stay for mental disorders than in public healthcare, where provider payments were made based on an annual global budget [23]. The modality of provider payments may be as important as insurance in determining the quantity of care.

In the African context, Chiromo has considerably shorter stays than most psychiatric hospitals, suggesting there is not an evident over-consumption of care. A general teaching hospital in Johannesburg approximates Chiromo with a mean psychiatric LOS of 15.4 days [24], but a readmission rate of only 7.5%, suggesting better continuity of care after discharge [25]. A public general hospital in South Africa, found a mean LOS for psychotic men of 43.9 days (sd 39.4) [26]. And a general public teaching hospital in Nigeria had a mean psychiatric LOS of 28.7 days (n = 371) [27].

In relation to components of care, Chiromo's ECT rate is high by global standards, but lower than among inpatient psychiatric populations elsewhere in Africa: it ranges from 21–28% in Malawi, Nigeria and South Africa [28]. The mean of 5 sessions approximates the 6 session dose recommended by the UK's National Institute for Health and Care Excellence (NICE) [29].

At 57.6%, occupancy rates were low at the time of study. One reason for keeping beds empty is that staffing capacity was not on par with infrastructural capacity, so that while the hospital could physically accommodate more patients, it could not do so to desired levels of quality. Another potential reason for low occupancy was the financial barrier to treatment.

With a per-capita GDP of Int$ 1,015 (Ksh 38,970) [30], treatment at Chiromo lies well beyond the reach of most Kenyans, making charge the main barrier to access. Indeed, Chiromo's occupational profile departs markedly from the national norm, with 50.6% of patients in formal employment, as compared to an average of only 9% of working age people (14% of those employed) [31]. By comparison, the World Health Organization's method for Choosing Programmes that are Cost-Effective (WHO CHOICE) estimates the “hotel cost” (personnel, capital, food) of an inpatient bed/day in a public, urban hospital in Kenya at Int$ 14.49 (Ksh 505) [32]. A comparable figure at Chiromo is the daily “bed fee” of Int $119 (Ksh 4500), considerably above the public sector estimate. Anecdotally, the lowest-income patients observed during the site-visit (eg someone employed lifting cement) were among those with employer-based insurance. Increasing insurance therefore may be a means of providing access to care from a broader population base.

If Chiromo patients have better outcomes than those in the public sector, the additional cost of care could be justified from an economic perspective. There are insufficient outcome data to rigorously compare; however some process indicators provide insight into the relative quality of care. The diagnostic profile at Chiromo mirrors that in Kenya's public psychiatric hospital, Mathari, where 34.4% have a substance use disorder; and 51.0% have schizophrenia or psychosis [33]. Chiromo's readmission rate of 22.2% is also in keeping with the that of 24.6% found in Mathari hospital [33]; however comparison on readmission is limited by the absence of published data on length of stay at Mathari.

A more meaningful comparison may be along the lines of human rights. In February 2011, CNN released a scathing documentary about Mathari [34] showing a dead body beside a live patient in an isolation cell. In response, the Kenya National Commission on Human Rights audited public mental healthcare, noting “systemic neglect” [35]. The Commission inspected three public psychiatric inpatient units, finding staff-to-patient ratios of 1∶80, and occupancy rates from a low of 105% to a high of 200%. Hygiene was poor, and hospitals lacked basic resources and equipment, like a functioning ECT machine. By comparison, Chiromo has a qualified nurse-to-patient ratio of 1∶20, hygiene is good, the pharmacy is stocked without shortage, and the ECT functions.

## Conclusion

Chiromo delivers acute psychiatric care each year to approximately 450 people, to quality and human rights standards higher than its public counterpart, but at considerably higher cost. If there were more efficient (lower-cost) ways of delivering care, Chiromo might expand its services and increase its occupancy. Means of lowering inpatient costs include: 1) using more generics; 2) shifting the mix in staff to reduce reliance on psychiatrists; and 3) reducing readmissions, possibly through developing more outpatient psychosocial interventions and intermediary care. Chiromo moved in this direction with the creation in 2012 of a half-way house for substance abuse; the same model could be applied for non-substance related psychiatric disorders. Incentives for cost-cutting could be built into insurance, for example by mandating the use of generics.

Our findings are limited by the absence of clinical outcome measures and of comparable data from the public sector. Comparison is also challenged by a lack of indicator of illness severity. Furthermore, generalizations on the overall quality of private versus public care cannot be made on the basis of this single case. Indeed, the Chiromo model is not currently generalizable, because its concentration of qualified professionals would be unsustainable at scale in this low-income setting. Nonetheless, this study is notable as a first analysis of insurance effects on mental healthcare in Africa, and provides a useful benchmark of private inpatient practice against which to measure alternatives and future change.
